# Surgical Anatomy

**Published:** 1850-05

**Authors:** 


*Surgical Anatomy.* By JOHN MACLISE, Surgeon. Part 2d. Lea & Blanchard. Philadelphia, 1850. Quarto, with fifteen colored plates.

In the January No. of the Examiner we took occasion to call the attention of our readers to this admirable re-print of a popular London publication. We now desire again to direct them to the second No. which has just made its appearance. It will be remembered that it is being published in numbers of quarto size, and in a uniform style; the plates in the present issue, therefore, are continuous with those in No. 1.

Plates 17, 18, and 19, exhibit the surgical dissection of the wrist and hand. Plates 20 and 21, the relative position of the cranial, nasal, oval and pharyngeal cavities, &c. Plate 22, the relative position of the superficial organs of the thorax and those of the abdomen. Plate 23, the relative position of the deeper organs of the thorax and those of the abdomen. Plate 24, the relations of the principal bloodvessels to the viscera of the thoracico-abdominal cavity. Plate 25, the relation of the principal bloodvessels of the thorax and abdomen to the osseous skeleton, &c. Plate 26, the relation of the internal parts to the external surface of the body. Plate 27, the surgical dissection of the superficial bloodvessels, etc. of the inguino-femoral region. Plates 28 and 29, the surgical dissection of the first, second, third and fourth layers of the inguinal region in connexion with those of the thigh.

From this analysis of the contents, it will be seen that the interest of those engaged in this particular department, cannot fail to be kept up by the admirable display of the surgical regions presented to them in these plates.

The representations are most faithful in their anatomical details, and in execution unequalled by any that have ever been brought before the profession in this country.

We again renew our thanks to the American publishers for their re-production, and for the reasonable rate at which they are offered to the medical public.

